# Subtrochanteric shortening osteotomy in adult sickle cell disease patients with cemented total hip arthroplasty for hip deformities secondary to childhood osteonecrosis: is healing a challenge?

**DOI:** 10.1007/s00264-024-06394-z

**Published:** 2024-12-21

**Authors:** Philippe Hernigou, Yasuhiro Homma, Claire Bastard, Byoung-Chol Yoon, Charles-Henri Flouzat Lachaniette

**Affiliations:** 1 Paris-Est Créteil University, Créteil, France; 2 Juntendo University Hospital, Tokyo, Japan

**Keywords:** sickle cell disease, hip osteonecrosis, childhood osteonecrosis, hip deformity, cemented stem, nonunion, subtrochanteric shortening transverse osteotomy

## Abstract

**Purpose:**

Hip deformity is frequent after childhood osteonecrosis in patients with sickle cell disease (SCD). When they are adults, they present a challenge as candidates for total hip arthroplasty (THA) because of abnormal bone development, their relative youth, and also because of their disease. Performing subtrochanteric osteotomy associated with THA is technically challenging, and healing of osteotomies has never been reported in this population with frequent osteonecrotic bone, whether using cemented or uncemented arthroplasties.

**Methods:**

We retrospectively analyzed 59 cemented THA with femoral corrective osteotomies (subtrochanteric shortening and transverse derotational osteotomy) performed on hip deformities between 1984 and 2018 in 59 sickle cell adult patients. The patient’s age at the onset of osteonecrosis was an average of 8.4 years (6 to 10 years), and at the time of the THA was 28.6 years (19 to 41 years). All the patients had a minimum followup period of six years. Endocrinopathy was frequently associated to SCD Data and consequences were evaluated on bone union. The mechanical variables, such as the length of the resected femur, limb lengthening, the location of the osteotomy site, the size of the stem bridging the osteotomy, and any complications, were also analyzed.

**Results:**

The average length of the resected femoral segment was 38.4 mm. The length of the femoral stem (bridging the osteotomy) was average 13 cm. The mean osteotomy union time was 10.6 months. Twenty-four osteotomies healed in six months, six in nine months, and 29 in twelve months, while five required bone grafts at nine months. The union time of the osteotomy was average 10.6 months. Complications included four cases of transient nerve palsy, and five intraoperative femur perforations. No statistically significant relationship was found between osteotomy union time and mechanical variables. The severity of endocrinopathy associated with sickle cell disease prolonged the healing time. In three cases, cement leakage into the osteotomy gap occurred without resulting in non-healing.

**Conclusion:**

Cemented THA, combined with a subtrochanteric femoral shortening with transverse derotational osteotomy, has a long union time but is effective for adult hip deformities of patients with sickle cell patients.

In children with sickle cell disease [1], hip involvement is quite common. This condition arises when the blood supply to the femoral head is disrupted, leading to the death of bone cells, which can result in pain and restricted movement. Unfortunately, when hip osteonecrosis occurs in very young patients (under 10 years old), it can cause anatomical distortions that impact growth and lead to deformities in adulthood.

Total hip replacement in these patients (Fig. 1) presents specific challenges influenced by bone anomalies, soft tissue issues, limb length inequality, and developmental abnormalities in the upper femur and acetabulum. Anatomical abnormalities may include the abnormal positioning of neurovascular structures due to soft-tissue contractures, misplaced hip center, limb-length discrepancies, a small acetabulum and femoral canal, and abnormal rotation of the proximal femur [1–2].

Several studies [3–5] have documented experiences with performing prostheses in severely dysplastic and high-riding hips with osteotomies. One crucial step in total hip arthroplasty (THA) is restoring the hip’s anatomical centre of rotation, which improves the function of the abductor muscles and provides long-term benefits. This osteotomy often involves both subtrochanteric femoral shortening and rotational change.

However, there are no studies on osteotomy healing in patients with sickle cell disease. Many surgeons believe that cemented stems carry the risk of cement leakage into the osteotomy gap, potentially causing nonunion at the osteotomy site. Nevertheless, cemented stem total hip arthroplasty (THA) with subtrochanteric transverse osteotomy offers some advantages for patients with sickle cell disease. In a femur weakened by necrotic bone, cemented stems provide more excellent stability than uncemented ones and significantly reduce the risk of intraoperative periprosthetic fractures, which are more familiar with uncemented stems. Additionally, cemented stem THA can be performed in most countries using conventional cemented stems. In contrast, uncemented stem THA may require specialized femoral stems, which can be challenging in certain regions where sickle cell disease is prevalent.

Therefore, this study aimed to explore the technical challenges and outcomes of subtrochanteric osteotomy for patients with hip deformities treated with cemented stems. Additionally, the study aimed to investigate any associations between osteotomy union time and clinical factors such as bone abnormalities related to sickle cell disease or cemented stem.

## Materials and methods

### Patients

We retrospectively reviewed the records of the 59 patients who underwent surgery between April 1984 and 2004. There is no funding source for this study. Human Ethics and Consent to Participate declarations are not applicable. The diagnosis of childhood osteonecrosis was determined through historical, clinical, and radiographic findings. The average age at the onset of osteonecrosis was 8.4 years (ranging from 6 to 10 years), and the average age at the time of THA was 28.6 years (ranging from 19 to 41 years).

Patients in this study were selected based on the following criteria: hip osteonecrosis before age ten, radiographic evidence of femoral deformities during childhood that could complicate the THA procedure and minimum five-year follow-up. No antecedent of infection was noted, and no bacteria was obtained from specimens obtained during surgery. The evaluation considered patients’ conditions [6], leg length, limp, and pain patterns. Surgeries on the opposite limb, such as epiphysiodesis, were recorded. Leg lengths were measured using a tape measure and blocks under the shorter limb to determine exact discrepancies and identify the length that best balanced the pelvis. A preoperative assessment of femoral and sciatic nerve function was conducted.

### Demographic data

The patient cohort consisted of 27 men and 32 women, with an average body mass index (BMI) of 23.6 kg/m² (ranging from 16.1 to 33.8 kg/m²). Most patients were homozygous for the sickle cell gene (haemoglobin SS), with three patients having hemoglobin S/hemoglobin C, and two having hemoglobin S associated with beta-thalassemia. The medical record review included transfusion documentation, chelation history, and recent laboratory values. Chronic transfusion was defined as receiving eight or more transfusions per year or at least one transfusion every seven weeks.

Risk factors for low bone mass [7–9] were observed during chilhood. We observed delayed growth and pubertal development, milk avoidance, lactose intolerance, deficiencies in bone-forming nutrients (Calcium, Vitamin D, Zinc), and low serum 25-OH vitamin D levels. Additionally, transfusion therapy can lead to iron overload and endocrine abnormalities. Growth failure [9–10] was characterized by a height Z-score lower than − 2.5 and/or the use of growth hormone therapy.

The need for ongoing thyroid hormone replacement therapy identified hypothyroidism. In females, hypogonadism was defined as being over 13 years old and not yet at Tanner B2, over 14 years old requiring estrogen replacement therapy, or over 15 years old with primary amenorrhea. In males, it was defined as being over 14 years old and not yet at Tanner G2, or over 17 years old and not yet at Tanner G4. Endocrinopathy was considered as dysfunction in at least one of three axes studied: growth failure, hypogonadism, or hypothyroidism.

### Medical status and preoperative management

Patients underwent a preoperative evaluation, as previously described, including a haematologic consultation. Blood products were matched for ABO Rhesus (Cc D Ee) and Kell antigens to prevent alloimmunization. Antibiotics were administered during surgery and for three days afterward. Implants were secured with antibiotic-loaded cement, and intraoperative cultures were obtained, all of which were negative.

### Radiographic evaluation of anatomic deformity

Preoperative radiographic assessments classified patients into four categories based on the severity of hip dysplasia. The minimum radiographic evaluations included anteroposterior pelvis and Lauenstein lateral radiographs. CT scans were used to determine hip versions accurately. Arteriography was used to visualize arteries and plan incisions to avoid vascular injury in complex THA procedures, especially in patients with altered vascular anatomy due to previous surgeries.

### Surgical technique

Patients were positioned laterally, and a curvilinear lateral incision was used for posterolateral exposure. The hip was dislocated posteriorly unless it was already severely subluxed or dislocated. The true acetabulum was exposed, and the acetabular cartilage was removed using a small reamer.

The decision to perform a subtrochanteric osteotomy with bone resection and derotation was based on preoperative planning and intraoperative evaluation of soft tissue tension during attempts to reduce the femoral head into the acetabulum. The procedure was evaluated by pulling the leg with the knee flexed to about 90° while maintaining consistent distal traction. While the trial stem was inserted into the proximal femur fragment, the trial femoral head was reduced into the cup. Traction was applied to the distal femur while the knee flexed at approximately 90°. The bony overlap length at the osteotomy site was excised from the distal femur (Fig. 2). On average, the excised femoral segment measured 38.4 mm, with a range of 33 to 45 mm. The osteotomy was made at an optimal level below the lesser trochanter, with the average osteotomy site being 21.1 mm (range: 15 to 30 mm) below the trochanter.

**Fig. 1:**
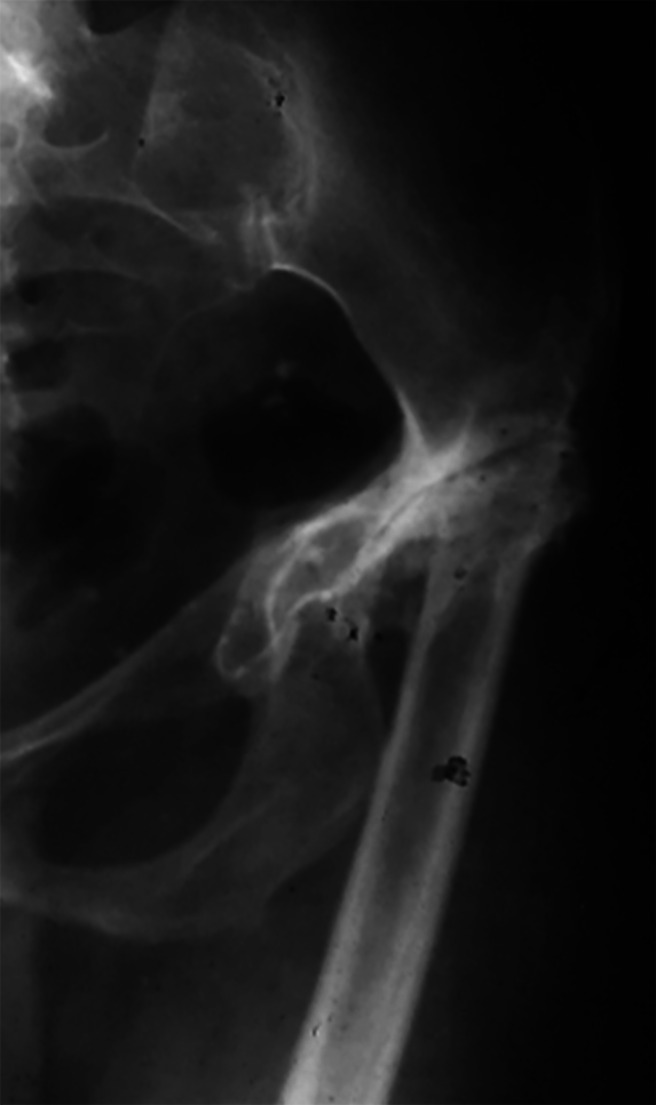
Abnormal anatomy of the proximal femur in a patient with SCD and hip osteonecrosis when 6 year old

**Fig. 2:**
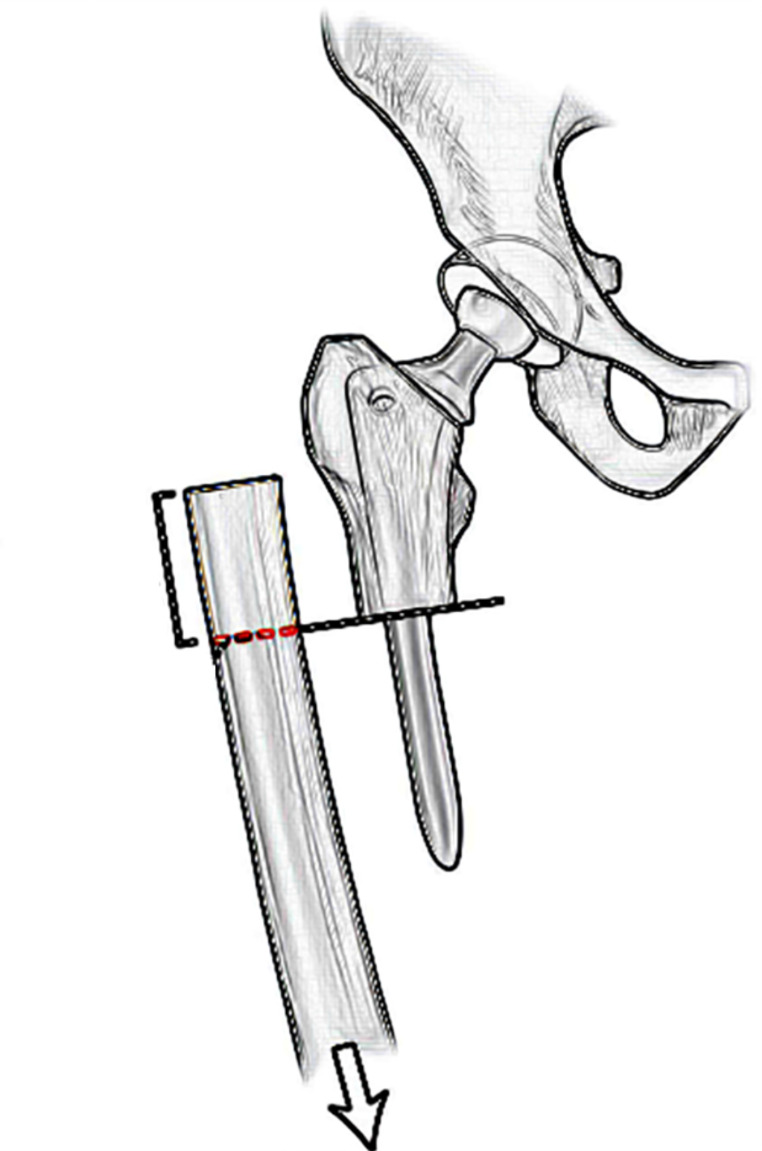
Measurement of the shortening during the surgery

Following this, the hip was prepared for the stem. Stems were cemented implants. The contact cuts were planarized both for the proximal and distal femur. Clamps and a transitory plate held both fragments in place to insert the cement and stem. Before cement was used, a rubber blade was temporarily wrapped around the femur to prevent cement from leaking through the osteotomy cut. The femoral component was inserted with the implant anteverted at 10–15°. During reconstruction, the anteversion of both femoral and acetabular components was assessed to maintain stability, aiming for a combined anteversion between 30° and 45°. Intraoperative assessment using the Ranawat Sign helped avoid stability issues.

### Statistical analysis

Statistical analysis employed chi-square tests, Student’s t-tests, and analysis of variance.

## Results

### Postoperative complications

#### Medical complications

These patients with SCD experienced significant blood loss, averaging 2,100 mL, requiring multiple transfusions for all patients. The increased blood loss was due to procedural challenges related to abnormal anatomy and necessary osteotomies.

Preoperatively, red blood cell exchange was performed in nine patients with severe acute chest syndrome or cerebrovascular episodes to reduce hemoglobin S levels below 30%. For others, the goal was to maintain postoperative haemoglobin levels above 8 mg/dL, targeting 10 mg/dL, using transfusion therapy. Despite these measures, the average postoperative haemoglobin level was 8 mg/dL, and three patients required intensive care due to massive intravascular haemolysis, with haemoglobin dropping below 4 mg/dL.

Two patients developed deep venous thrombosis.

#### Surgical complications

**Fig. 3:**
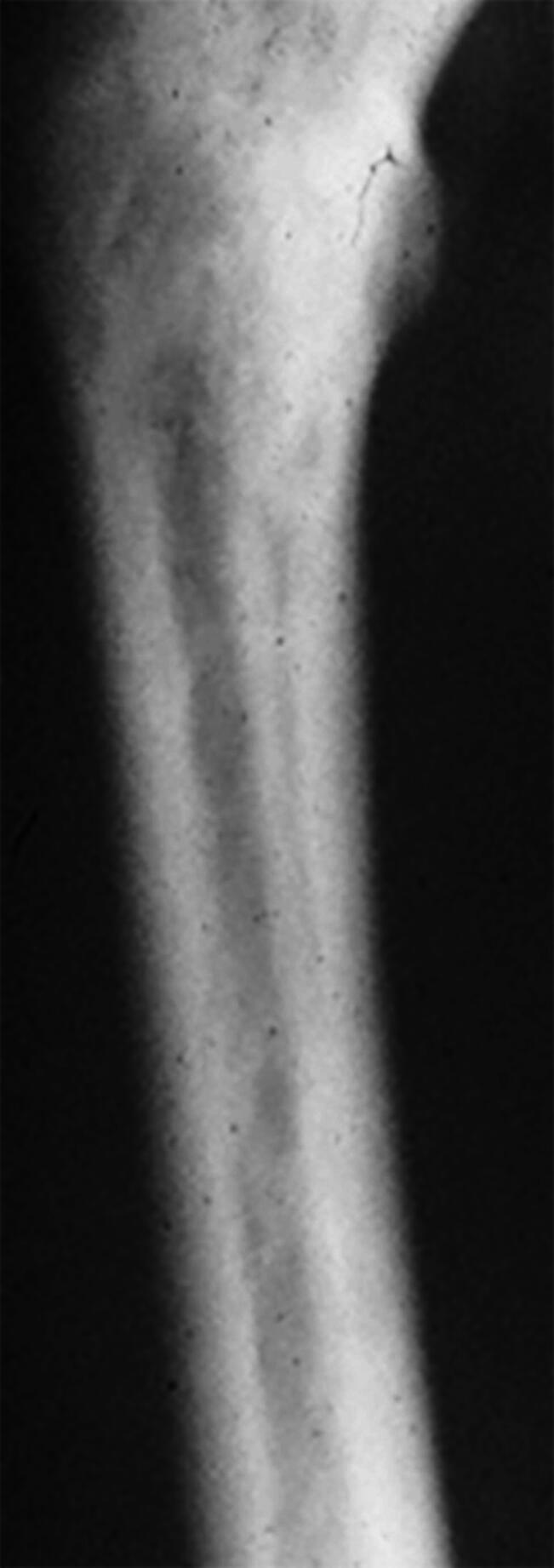
Dense bone sclerosis in the medullary canal

Due to osteonecrosis, dense bone sclerosis (Fig. 3) and medullary canal obliteration were found in 35 femurs, requiring reaming. Femoral perforation occurred in five patients, with three needing additional plates and screws. Excessive bleeding led to wound haematomas and prolonged (5 days instead of 2 days) drainage in four patients.

Early dislocation occurred in 3 hips, with no recurrent or late dislocations. Heterotopic ossification developed in ten hips: six rated Class 1, four Class 2, and two Class 3 per Brooker et al. Other complications included four transient sciatic nerve palsy.

Iterative surgery to graft the osteotomy site was necessary in five patients. The indication for grafting was an absence of callus at nine months followup. Graft was obtained from iliac crest.

### Osteotomy healing

Twenty-four femoral osteotomies achieved healing within six months, six achieved Union within nine months, 31 within twelve months, and five required bone grafts at nine months. The mean osteotomy union time was 10.6 months (7.6 to 18 months).

The distance from the osteotomy site to the lesser trochanter was an average distance of 21.1 mm (ranging from 15 to 30 mm). The mean length of the stem bridging the osteotomy was 13 cm (ranging from 12 to 15 cm). The shortening length (excised femoral segment) was average 38.4 mm, varying from 33 to 45 mm. The average stem length extended 9.2 cm below the osteotomy site. Mechanical factors, such as the length of femoral resection, limb lengthening, and the distance between the osteotomy site and the lesser trochanter, were not found to be risk factors for healing. In three cases, radiographic examination revealed some cement leakage into the osteotomy gap associated with the callus.

Sickle cell disease (SCD) appears to prolong osteotomy union time (Table 1), particularly in patients with endocrinopathy involving dysfunction in at least one of three studied axes: growth failure, hypogonadism, or hypothyroidism. Thirty-six patients received chronic transfusions, and 32 had low serum 25-OH vitamin D levels. Twenty-six patients exhibited at least one of the following conditions: growth failure, diabetes, hypogonadism, or hypothyroidism. All patients received routine medical care for their primary haematological condition.

**Table 1:** influence of some factors on healing of the osteotomy

Factors Related to healing before 6 months	healing	non-healing	p
*N* = Nb (percentage = N/59)	24 (41%)	35 (59%)	
Gender (Male) = 27	10	17	NS
Female = 32	14	18	NS
Hypothyroidism *N* = 11	1	10	0.04
Hypogonadism *N* = 19	2	17	0.04
Initiated Chelation Therapy ≥ 6 years *N* = 25	3	21	< 0.01
Height Z-score ≤ − 2.5 *N* = 26	6	20	0.02
Transfusion Duration, years > 14 years *N* = 30	5	25	< 0.01
Chelation Duration, years > 10 years *N* = 28	4	24	< 0.01

**Fig. 4:**
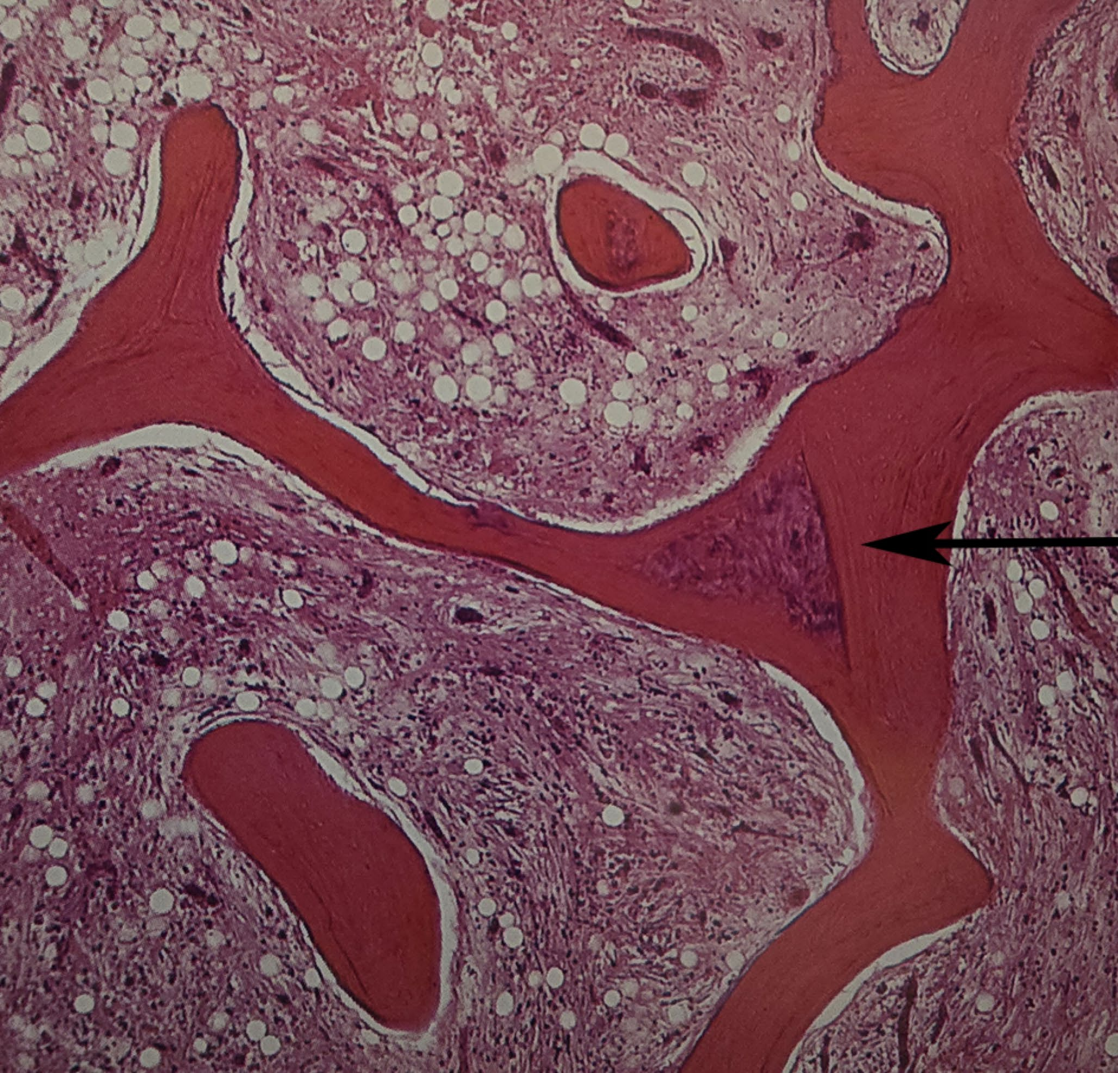
In the centre of the callus wich lies within a vascular portion of the bone marrow of the callus is a purple triangular (arrow) segment of necrotic woven bone (H and E x 30). In this callus, infarction occurs again and again. Revascularisation will repair the callus infact in many instances. The final histologic feature reflect this sequence of multiple infarcts occurring in the same anatomic site

**Fig. 5:**
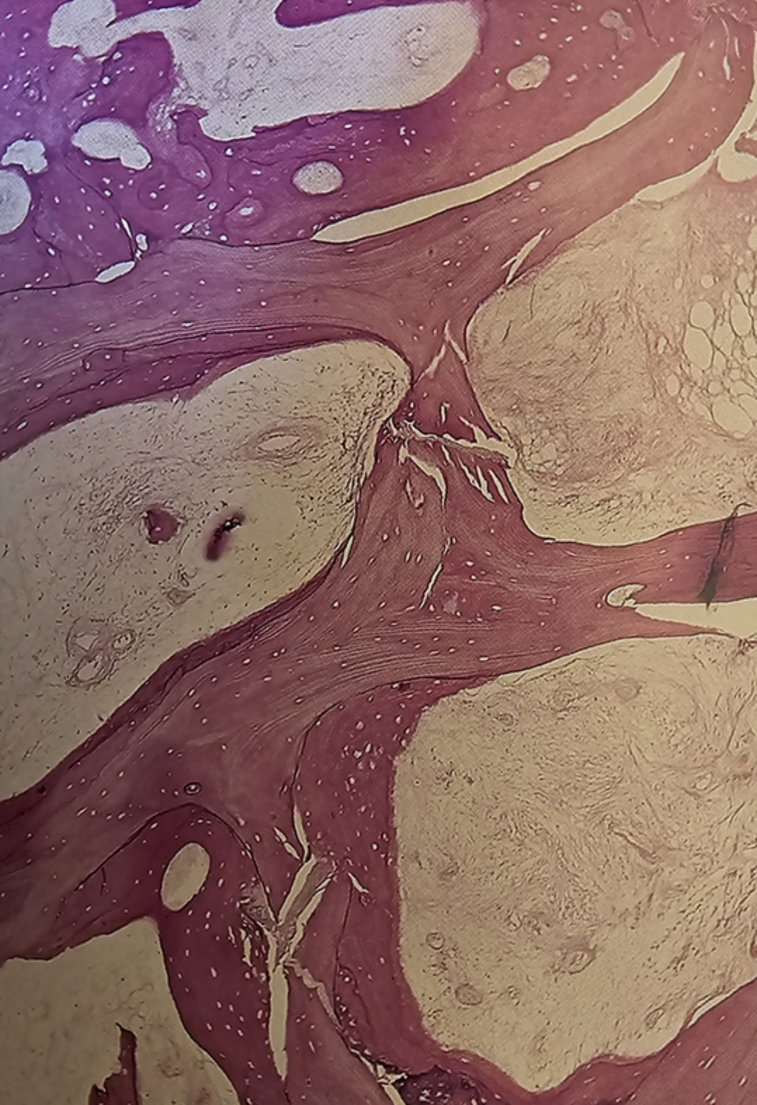
Detail of cortex and callus showing necrotic elements with no cellular outlines or blood vessels. The bone has been reinforced, but all the bone original and repair is now necrotic

**Fig. 6:**
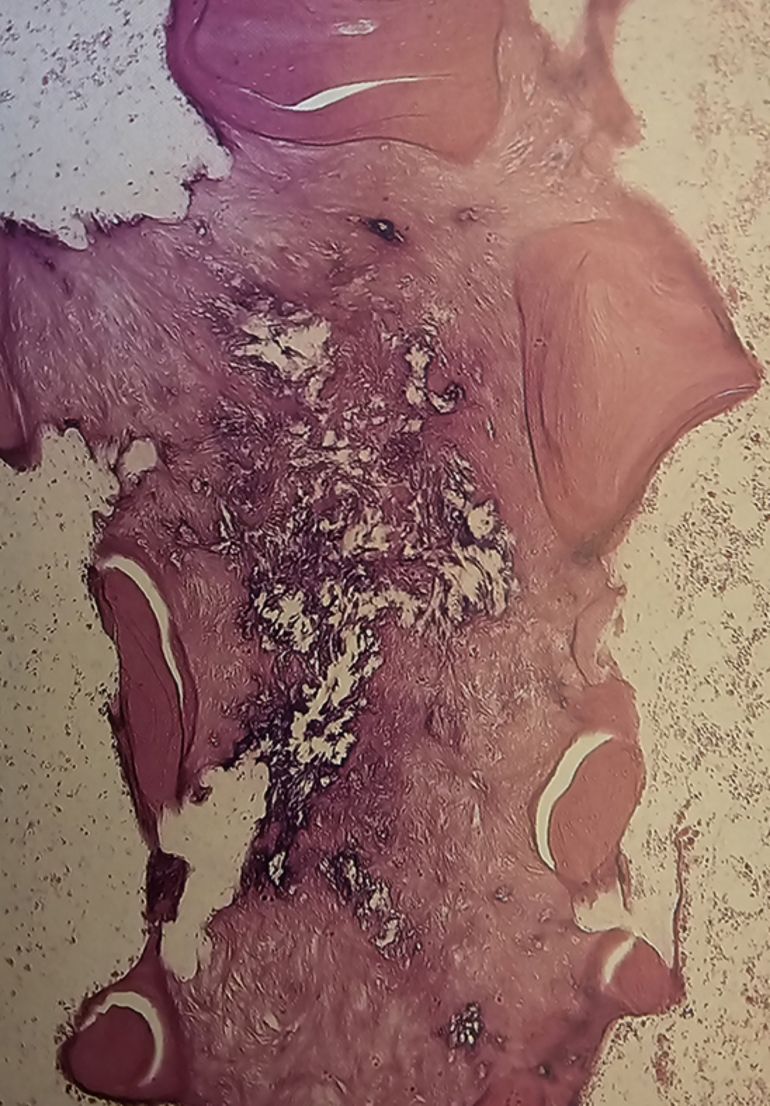
Detailed of calcified fat between fragments of several bone trabeculae. All tissues in this photograph (bone and bone marrow) is necrotic

**Fig. 7:**
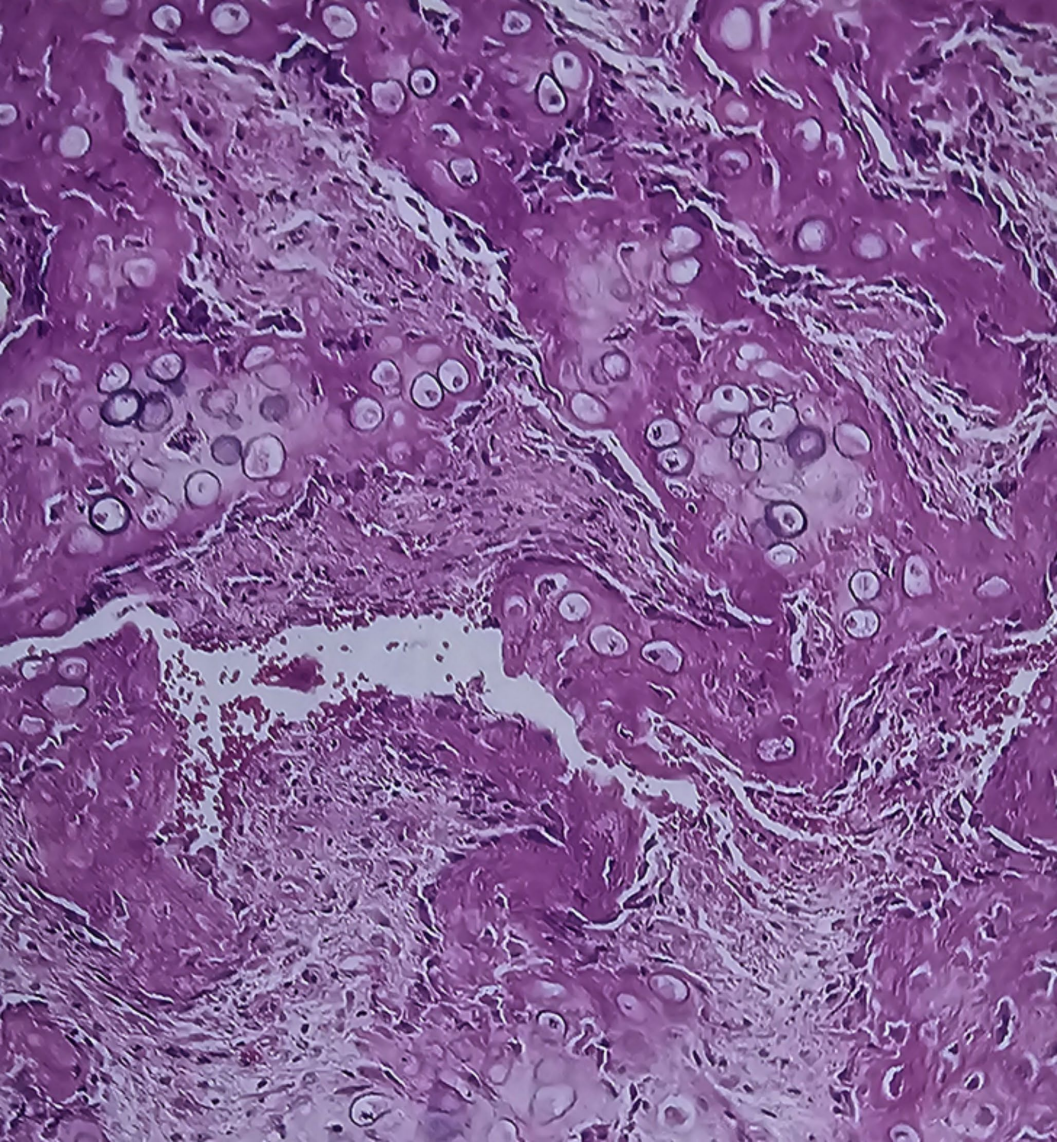
The picture shows tissue that is neither bone nor cartilage. both a cartilaginous matrix with chondrocytes and new osteoids with numerous osteoblasts are juxtaposed in a single trabecculum

For the five patients who had a bone graft from the iliac crest at the osteotomy site due to the absence of evidence of radiologic healing at nine months, biopsies were obtained from the osteotomy site. In these five patients with sickle cell disease (SCD), the formation of a callus after an osteotomy follows a somewhat different trajectory than in individuals without the disease due to evident vaso-occlusive episodes in the callus. Bone healing was delayed, and histologically, the initial formation of woven bone (the primary callus) was slow and poorly organized (Figs. 4, 5, 6 and 7) due to iterative frequent vaso-occlusive crises, and signs of ischemic necrosis or infarction in the callus. The necrotic bone areas appear as empty lacunae with surrounding devitalized bone matrix (figures).

## Discussion

### Medical complications and transfusions

Childhood osteonecrosis [11] of the hip in patients with sickle cell disease (SCD) often results in significant anatomical deformities that require complex surgical interventions in adulthood, more frequently on the femoral side [12]. One of the most immediate complications for SCD patients undergoing total hip arthroplasty (THA) is significant blood loss, requiring multiple transfusions for all patients [13]. This series typically experiences higher blood loss than SCD patients without hip deformities, with an average loss of 2100 mL versus 950 mL, respectively in our experience [14], as in the literature [15–16]. The increased blood loss is primarily due to the technical difficulty of the procedure, caused by abnormal anatomy and the need for osteotomies. Additionally, frequent childhood transfusions elevate the risk of transfusion reactions, alloimmunization, and difficulty in obtaining enough blood for surgery. We aimed to maintain a postoperative hemoglobin level above 8 mg/dL, aiming for 10 mg/dL, by employing straightforward perioperative and postoperative transfusion therapy. Despite these efforts, achieving a postoperative hemoglobin level of 10 mg/dL was challenging, with the average level being 8 mg/dL.

### Cemented stems for subtrochanteric osteotomy in SCD

Although cemented fixation in total hip arthroplasty is becoming less common, even in sickle cell patients, there remains a strong indication for its use, particularly in complex surgeries such as those involving subtrochanteric osteotomy. In patients with poor bone quality, especially in cases where the femur has been altered during childhood due to epiphyseal or metaphyseal necrosis. In cases of sickle cell disease, where the cortical bone is thinned, the bone is spongy and weak due to bone marrow abnormalities, or osteonecrosis, uncemented fixation poses significant risks. These include distal migration of the implant or fractures, even with minimal trauma or rotational forces.

The success of osteointegration, by definition, relies on the quality and integrity of the bone tissue and the shape of the medullary canal—both of which are often significantly compromised in these patients. However, uncemented THA might offer advantages by avoiding complications associated with cementing in the presence of cardiac disease in this population [17]. A relevant concern is whether the use of cement in fragile patients can lead to specific complications, such as fat embolisms. These embolisms, though rare, are particularly dangerous in patients with cardiological issues, which can occasionally occur in sickle cell patients. While this complication has been observed in femoral cementing, it is now infrequent in scheduled surgeries where patients are thoroughly assessed and treated under optimal conditions. Most of these patients undergo comprehensive cardiological evaluations, including ultrasound. In cases where an open foramen oval is detected, a discussion may arise about whether to close the foramen before proceeding with the cemented prosthesis.

Traditionally, rasping the femur in cemented hip arthroplasty is used to gauge the size of the femoral canal and establish a sufficient cement mantle. Typically, this process offers the surgeon tactile feedback, allowing for precise sizing of the femoral canal and ensuring an appropriate cement mantle thickness. However, this can be challenging in SCD, as the tactile sensation differs during reaming, with a risk of femur perforation. After reaming, the endosteal canal becomes round. Based on senior experience, after reaming, it is better to use a larger stem to achieve partial contact with the bone, minimizing the risk of rotation, with cement filling the remaining space.

### Analysis of bone healing at the osteotomy site

Despite the global prevalence of sickle cell disease in the world, only one article reports 59 fractures in these patients [18], without specifying consolidation time or the risk of pseudarthrosis. Osteotomy union time was prolonged in this series of SCD patients, especially when patients had endocrine dysfunction in at least one of three axes (growth failure, hypogonadism, or hypothyroidism), compared to the healing time of similar osteotomies in literature reports when absence of SCD. Evidence suggests that the stem length bridging the osteotomy site is crucial for stability.

Various factors, such as the length of femoral resection, limb lengthening, and the proximity of the osteotomy site to the lesser trochanter, were not identified as significant risk factors. The average stem length extended 9.2 cm below the osteotomy site. During the procedure, it was crucial to secure both femoral fragments with bone clamps to minimize the risk of cement leakage into the osteotomy gap. Evidence indicates that the femoral stem bridging the osteotomy site is essential for stability, though recommendations on the ideal length vary. Ozan et al. [19] suggested the stem should extend 4–5 cm beyond the osteotomy, while Yang et al. [20] proposed 3 cm as adequate. Kawai et al. [21] proposed 7 cm below the osteotomy for the stem, acting as an intramedullary nail and enhancing stability. In our study, the average stem length bridging the osteotomy site was 9.2 cm (range: 76.5–106.2 mm), slightly exceeding the lengths suggested by previous studies.

In conclusion, cemented THA, combined with a subtrochanteric femoral shortening with transverse de-rotational osteotomy, has a long union time but is effective for adult hip deformities of patients with sickle cell patients.

## Data Availability

No datasets were generated or analysed during the current study.
